# ASO Author Reflections: A Path Forward: Improving Access to Guideline-Concordant Treatment for Foregut Cancers

**DOI:** 10.1245/s10434-024-15836-2

**Published:** 2024-07-26

**Authors:** Annabelle L. Fonseca, Smita Bhatia, Martin J. Heslin

**Affiliations:** 1 Department of Surgery, The University of Alabama at Birmingham, Birmingham, AL USA; 2 Institute for Cancer Outcomes and Survivorship, The University of Alabama at Birmingham, Birmingham, AL USA

## Past

Foregut cancers account for over 20% of new cancer deaths in the United States each year. These cancers have poor overall survival rates due to inherent biological aggressiveness, relative chemoresistance, and significant challenges in the delivery of cancer care. Despite evidence that guideline-concordant treatment (GCT), i.e., stage-specific, standard-of-care treatment, improves patient outcomes in foregut cancers, a large proportion of patients with foregut cancers do not receive GCT.^1^ Numerous institutional and administrative database studies have shown that sociodemographic variables such as race, socioeconomic status, and rurality are predictive of GCT, however these studies lack detailed insights into individual barriers preventing access to GCT. Non-receipt of GCT is likely multifactorial, and a result of multiple, unmeasured barriers along the cancer care continuum, several of which may be potentially modifiable.

Our study aimed to identify specific barriers to the receipt of GCT among patients with foregut cancers treated at an academic center in the Deep South through a detailed review of electronic health record (EHR) data. Using a root cause analysis approach, we sought to explore the underlying issues contributing to non-receipt of GCT in order to provide a comprehensive understanding that could inform subsequent targeted interventions to improve cancer care equity.

## Present

Our study delves into the multifaceted barriers preventing receipt of GCT.^2^ Detailed abstraction of data from EMRs and a structured root cause analysis approach demonstrated multiple contributory and often intersecting patient, physician, institutional environment, and broader system-related factors. Patient factors included transportation and financial barriers, lack of social support, health literacy, self-advocacy, health avoidance, comorbidities, and deconditioning, while physician factors included time and resource constraints, medical education, and decision making, as well as bias and nihilism. Institutional environment factors included lack of automated systems, understaffing, lack of outpatient access, and lack of patient-centric services such as prehabilitation programs, multidisciplinary clinics, and patient support services. Broader system factors included insurance-related factors (lack of insurance, inadequate insurance coverage, high out-of-pocket costs), limited healthcare access (primary care and subspecialty care), and public policy and infrastructure, including inadequate public transport, Medicaid non-expansion, and prison healthcare.

While addressing these intersecting factors comprehensively requires policy-level solutions, we proposed several institution-level solutions to improve access to GCT among patients with foregut cancers. Implementing automated systems at the institutional EHR level could improve access to GCT by automating scheduling, thereby reducing loss to follow-up after missed appointments, and improving compliance at the physician level with prompts for appropriate staging and referrals to relevant oncologic expertise. Institutional prioritization of staffing at all patient-facing levels, including scheduling, clinic staffing, social work, care coordination, and navigation would not only improve patient experience overall but also improve cancer care equity for patients most at risk of non-receipt of GCT. Other institution-level solutions proposed included co-location of multidisciplinary cancer clinics and expansion of telehealth capabilities.

## Future

Future steps towards equitable cancer care must build upon our current understanding, while incorporating innovative methodologies. Individual institutions should strive to become Learning Health Systems, continuously analyzing their own data and integrating them with best practice to continuously improve institutional clinical practice and patient outcomes. Advanced technologies such as artificial intelligence and machine learning could be leveraged to prospectively identify patients at risk for non-GCT, allowing early intervention and targeted support.

While the same solutions may not yield the highest impact across different institutions and geographic locations, integrating datasets from multiple institutions may allow the identification of patterns specific to different populations and healthcare settings, ensuring the development of interventions that are tailored to the communities they serve. Multi-site studies may allow us to understand the most important factors in various environments, thus guiding the development of effective and impactful interventions across diverse settings.

There is also a critical need for policy-level interventions. Medicaid expansion has been associated with improved access to cancer care and improved outcomes among patients with foregut cancers.^3,4^ Broader adoption of Medicaid expansion would not only improve patient access to GCT but could provide an avenue for institutions to fund interventions aimed at improving access to GCT. Medicaid expansion has the highest impact on safety-net hospitals^5^ and could provide the critical financial support to expand patient-centered services at these institutions. Recent updates to the Centers for Medicare & Medicaid Services’ Physician Fee Schedule includes new Principal Illness Navigation (PIN) and Community Health Integration (CHI) codes that reimburse clinical teams for complex care and social determinants of health (SDOH)-related navigation. Value-based payment models that evaluate clinicians on the quality and cost of care delivered may also consider how SDOH-related navigation could be incentivized and reimbursed. Future research and interventions must account for the evolving healthcare landscape and the unique needs of diverse patient populations to ensure high-quality, equitable cancer care.
